# NSP-SCD: A corpus construction protocol for child-directed print in understudied languages

**DOI:** 10.3758/s13428-024-02339-x

**Published:** 2024-02-15

**Authors:** Sonali Nag, Sunila John, Aakash Agrawal

**Affiliations:** 1 Department of Education, University of Oxford, Oxford, UK; 2 Department of Speech and Hearing, Manipal College of Health Professions, Manipal Academy of Higher Education, Manipal, India; 3 NeuroSpin, CEA, Gif-sur-Yvette, France; 4 The Promise Foundation, Bangalore, India

**Keywords:** Written language, Child-directed print corpus, Lexical diversity, Akshara, Phoneme length

## Abstract

Child-directed print corpora enable systematic psycholinguistic investigations, but this research infrastructure is not available in many understudied languages. Moreover, researchers of understudied languages are dependent on manual tagging because precise automatized parsers are not yet available. One plausible way forward is to limit the intensive work to a small-sized corpus. However, with little systematic enquiry about approaches to corpus construction, it is unclear how robust a small corpus can be made. The current study examines the potential of a non-sequential sampling protocol for small corpus development (NSP-SCD) through a cross-corpora and within-corpus analysis. A corpus comprising 17,584 words was developed by applying the protocol to a larger corpus of 150,595 words from children’s books for 3-to-10-year-olds. While the larger corpus will by definition have more instances of unique words and unique orthographic units, still, the selectively sampled small corpus approximated the larger corpus for lexical and orthographic diversity and was equivalent for orthographic representation and word length. Psycholinguistic complexity increased by book level and varied by parts of speech. Finally, in a robustness check of lexical diversity, the non-sequentially sampled small corpus was more efficient compared to a same-sized corpus constructed by simply using all sentences from a few books (402 books vs. seven books). If a small corpus must be used then non-sequential sampling from books stratified by book level makes the corpus statistics better approximate what is found in larger corpora. Overall, the protocol shows promise as a tool to advance the science of child language acquisition in understudied languages.

**Supplementary Information:**

The online version contains supplementary material available at 10.3758/s13428-024-02339-x.

Children’s books have a wide-ranging influence on child development. Their power is considered to lie in the language that books carry because this written language is typically more varied and complex in contrast to spoken language. According to an estimate that used English material, child-directed print carries two-and-a-half times more word types and three times more rare words than child-directed conversational speech (Massaro, 2015). In addition to more diverse and rare words, the language encountered in child-directed print has a larger proportion of longer, more abstract, and more morphologically complex words that are also often later acquired in development than the words found in child-directed speech (Dawson et al., 2021). It is not surprising then that books are a rich resource for enhancing children's language, literacy, cognitive and socio-emotional skills (e.g., Grolig et al., 2019; Kara-Soteriou & Rose, 2008; Mol & Bus, 2011; Nation et al., 2022; Parry et al., 2014). Book corpora have also been used as a comparison set for mapping early spoken production (Montag & MacDonald, 2015; Saiegh-Haddad & Spolsky, 2014), early language exposure at home (Hayes, 1988) and in school (Schleppegrell, 2001; Shu et al., 2003), and to draw out the characteristics of a writing system (Nag, 2014).

## Characteristics of child-directed print corpora

The majority of available child-directed print databases are concentrated within American and British English, European and Brazilian Portuguese, French, German, Greek, Italian and Spanish (e.g., Berber Sardinha et al., 2013; Carroll et al., 1971; Corral et al., 2009; Lambert & Chesnet, 2001; Lete et al., 2004; Marconi et al., 1993; Martinez & Garcia Perez, 2008; Masterson et al., 2010; Schroeder et al., 2015; Soares et al., 2014; Terzopoulos et al., 2017). A small number of other languages also have a growing database of child-directed print, notably Chinese (Huang et al., 2020; Li et al., 2022; Shu et al., 2003; Xing et al., 2004) and Turkish (Aydin, 2019; Tolgay, 2015). These databases provide excellent examples of source materials for corpora construction. In addition, many of these language corpora have gained from automatized parsers that exponentially decrease computation time. Print included in these corpora may represent different book types (storybooks, information books) and genres (rhymes, narrative texts). These books may be bestseller titles on popular websites or high in circulation statistics in libraries. Teacher-, librarian-, parent-, and child-recommended lists also provide the material.

Corpora differ in size. For example, in English, corpora in influential studies have ranged from 57,000 words from 112 picture books (Massaro, 2015) to 68,103 words from 100 picture books (Montag, 2019; Montag et al., 2015); 319,435 words from 160 fiction books (Dawson et al., 2021; Hsiao et al., 2022); 698,286 words from 40 texts of imaginative fiction (Thompson & Sealey, 2007) and 2.4 million words from 1708 fiction and nonfiction documents (Montag & MacDonald, 2015). Examples of substantial corpora sizes in other European languages include 10 million words from 500 German books (Schroeder et al., 2015), 4.1 million words from 155 Norwegian children’s books (Dyvik et al., 2016) and 1.3 million words from 116 Greek textbooks (Terzopoulos et al., 2017). The range is similarly big in Chinese (e.g., Li et al., 2022: 22 million words from 2131 curricular and extracurricular books; Shu et al., 2003: 2570 characters from 12 elementary textbooks; Huang et al., 2020: 2.65 million characters and 1.83 million words from 52 textbooks and 43 storybooks) and Turkish (Aydin, 2019: 450,000 words from 268 narrative texts; Tolgay, 2015: 22,274 words from 536 books for 3-to-5-year-olds). These sizes reflect the aspiration in science to develop a larger corpus because estimates for some properties of the language are sensitive to corpus size such as the estimates for lexical diversity. An empirical prediction, called the Herdan–Heaps law, is that the relationship between types and tokens changes non-linearly with sample size (Heaps, 1978; Herdan, 1960). Specifically, as sample size increases, the number of types initially grows rapidly but eventually levels off, since words begin repeating more often in larger samples. This causes the type-token ratio (the number of types divided by total tokens) to decrease as sample size increases. The Herdan–Heaps law has been mathematically formalized to model this curve and provides crucial context for comparing lexical diversity, or the representative ‘vocabulary’, across different sample sizes. For our purposes, the concern is that smaller corpus sizes, particularly below a certain threshold, not only produce type-token ratios that are not a clear and actual representation of a child's learning environment, but then also place limits to cross-language comparison of lexical diversity.

Corpora also differ in the strategy used for corpus construction. A common method is to purposively select materials (e.g., school textbooks; children’s books: see descriptions above). A random selection of books from a larger collection is another approach but this is rare (see Sütçü, 2022). In both of these approaches, all sentences are included in the corpus with unaccounted sources of bias in lexical analysis managed through a post hoc random sampling of a pre-specified selection from the corpus. Multiple simulations are then executed within each sample size (e.g., Montag et al., 2015: randomly sampled 100 to 68,100 word sizes; Dawson et al., 2021: 100 to 50,000 word sizes). Such within-corpus multiple sampling can ‘detect changes within a text as well as differences between texts’ (Covington & McFall, 2010). The sensitivity advantage conferred by a random sampling algorithm could potentially be extended to corpus construction itself where, rather than a post hoc random sampling, the corpus is constructed through selective sampling of materials from multiple books. We did not find any study examining this approach and will examine the approach in this study using a cross-corpora analysis.

## Understudied languages and language characteristics

Child-directed print corpora provide the opportunity for systematic psycholinguistic research. For example, at the lexical level, a corpus can be used to examine word characteristics such as their frequency, phonological length, orthographic characteristics, and parts of speech (PoS). Other metrics include lexical density (the proportion of select PoS categories such as nouns, lexical verbs, adjectives, and adverbs to the total number of words), lexical diversity (the count of word types and type-token ratio estimated either as a simple ratio or using multiple randomly selected token sizes), and lexical sophistication (the proportion of rare words to the total number of words in a text). At the syntactic level, estimates of the linguistic properties of book text can be related to the morphological structure of words and the frequency of sentences with or without certain syntactic units, for example, subject-predicates, questions, imperatives, copulas, complex lexical verbs, passives, and relative clauses. An understanding of these features using corpus statistics can contribute to more informed measurement of and intervention for language and literacy learning, especially in understudied languages (e.g., Arabic: Schiff & Saiegh-Haddad, 2017; Malay: Lee & Lee, 2021).

Child-directed print corpora are also an excellent research tool to specify language-specific psycholinguistic patterns. For instance, in Turkish, the frequency of function words (e.g., conjunctions, prepositions), adverbs and multifunction words are noted to be higher than other lexical categories while at the level of suffixes, the frequency of inflectional suffixes (dative, past tense and nominal suffixes) is higher compared to derivational suffixes (Sütçü, 2022). Similarly, multisyllabic words, function words such as pronouns and deictic words such as ‘this’ and ‘that’ are higher than monosyllabic words in a 27,672-word Malay print corpus from five primary school textbooks (Lee et al., 2012). These language characteristics stand in contrast to the much-studied English, which is morphologically sparse, with fewer function words and more monosyllabic words. The study of ‘translation universals’ is another example of an understudied language characteristic that potentially defines the book language children encounter. A syntactic comparison of a print corpus comprising 40 original Finnish children's books and 40 English-to-Finnish translated books shows less colloquial words but more non-finite constructions and certain types of conjunctions in translated texts (Puurtinen, 2003). It appears that translated children’s materials have striking psycholinguistic features that are not necessarily found in the original language.

The focus of this paper is another understudied language - Kannada, a Dravidian, agglutinative language spoken in the southwestern part of the Indian peninsula by approximately 48.6 million native speakers (Kannada Ethnologue, 2022). Kannada is an official, administrative language of the state of Karnataka with a classical language status in India. It is a morphologically rich language characterized by multisyllabic words and a generous use of suffixes. The Kannada orthography is alphasyllabic with orthographic units called *akshara*. The akshara may be categorized by type (Nag, 2017), including units of consonants with an inherent vowel (Ca), consonants with other vowels (CV), and more than one consonant paired with or without a vowel (CC, CCa, CCV, CCCa, CCCV). Until recently, only two psycholinguistically analyzed corpora were available in Kannada. The first, an adult-directed prose of 100,000 words (Ranganatha, 1982) and the second, a child-directed print corpus comprising 8549 words from 101 reading cards (Patel, Bapi, & Nag, 2013, cited in Nag, 2014). The construction of a new child-directed print corpus of 150,595 words from 402 books (Nag, Nagendra, et al., 2021a) provided the database for this study.

## Motivation for this study

Although child-directed print corpora can assist researchers and teachers alike in selecting material that closely reflect language-specific characteristics, such research infrastructure is not available in most of the world’s languages. One reason may be because the language does not yet have widespread publishing of children’s materials. Another reason for missing corpus tools is that corpora development requires time. For spoken corpora, the transcription of the spoken data has been reported to be ten times the time taken for the original audio recording of the spoken language (MacWhinney, 2000). While print corpora do not begin with audio recordings, transcription may still be needed when the script is not widely known and conversion to a known script increases accessibility (e.g., transcribing Indic scripts in the Latin script). Moreover, additional time is needed for manual psycholinguistic coding when precise natural language parsers are not yet available. One plausible way forward is to draw upon materials becoming available in understudied languages but limit the intensive psycholinguistic work to a small-sized corpus. There is, however, little systematic enquiry about how to make robust small-sized corpora. The current study examines the potential of a protocol for construction of a short, non-sequentially sampled, child-directed print corpus through a cross-corpora analysis (Study 1) and a within-corpus analysis (Study 2).

We examined two research questions. First, is there equivalence in word-level characteristics between a larger corpus and a corpus based on a non-sequential sampling protocol for small corpora development (NSP-SCD)? For this, we used cross-corpora analysis to compare lexical diversity and word length, and orthographic diversity. We hypothesized that there will be no difference on these characteristics between the non-sequentially sampled small corpus and the larger corpus. As a further robustness check we examined the characteristics of the said corpus when the method of selection of a token size for generating type-token estimates was either random or sequential. Here we hypothesized that the lexical characteristics of the non-sequentially sampled small corpus would not change whether token selection was random or sequential because the known likelihood of sequential material capturing more word repetitions (Montag et al., 2018) would already be controlled in the NSP-SCD. Finally, we examined corpora characteristics when the comparison set is a same-sized, small corpus but constructed sequentially by taking continuously appearing sentences.

The second research question is whether corpus statistics in the shorter non-sequentially sampled corpus change across book levels. We hypothesized that books for older children will have a developmental increase in text demand and so, the linguistic complexity at the lexical, orthographic, and phonological levels would increase with an increase in book levels. In Study 2, a within-corpus analysis was used to examine this with the smaller corpus.

## Method

The Promise Foundation Corpus of Child-Directed Print in Kannada comprises 24,243 sentences and 150,595 words drawn from 402 books (Nag, Nagendra, et al., 2021a). The books in this corpus (henceforth the larger corpus) are commonly read to and read by children between 3 and 10 years and include award-winning and bestselling books, and books recommended by parents, teachers, and librarians, and published in or before the year 2020. Book types include story collections, folktales, non-fiction, translated works, textbooks, picture books and chapter books. Each book is categorized by book level based on four parameters (Padilla et al., 2021): book content (focusing on the theme, concept load, demand on background knowledge and its potential to interest children); organization of ideas (its coherence, clarity and concreteness); book language (including the vocabulary, length and complexity of sentences and individual words, and use of figurative language); and design (features such as layout, typography, inter-word and inter-sentence spacing, text on page, and supporting illustrations). Of the 402 books in the 150,595 word corpus, 109 are tagged as appropriate for 3-to-5-year-olds, 199 for 6-to-8-year-olds, and 94 for 9-to-10-year-olds (comprising 17,818, 67,583, and 65,194 words, respectively). Individual book lengths range from 7 to 1754 sentences and every book and sentence in the corpus is traceable through a unique identifying number.

A small corpus was drawn from the larger corpus using a sampling frame developed for constructing small corpora in Asian languages (Nag, Dulay, et al., 2021b). For books with fewer than ten sentences, one sentence from the book was randomly chosen. For books with more than ten sentences but less than 32 pages, every tenth sentence was extracted. To manage biases in language sampling, sentence selection began from one of the first three sentences identified through a random number generator. Every tenth sentence from the chosen sentence number was then selected (e.g., sentence 1, 11, 21…, or 2, 12, 22…, or 3, 13, 23...). The industry standard for young children’s books is 32 pages and between 500 and 600 words. In order to manage the resource-intensive nature of manual parsing, for those books with more than 32 pages, a text track of 1500 words was randomly selected, the starting sentence was randomly picked from one of the first three sentences within this tract, and the sentence selection rule of every-tenth-sentence was applied.

This selectively sampled 2661 sentences with 17,431 words (John et al., 2021), is the shorter corpus examined in this study. In Study 1, all words in the shorter corpus (henceforth the non-sequential small corpus) were examined against the larger corpus (a cross-corpora analysis). Given our interest in mapping language-specific characteristics, sentences from 402 books in the non-sequential small corpus was further analyzed in Study 2 (a within-corpora analysis). For this, each word was tagged for parts of speech within the sentential context it appears.

## Study 1: Cross-corpora analysis

To test the first research question and to study the robustness of a smaller-sized corpus, the nature of the 17,431-word tokens in the selectively sampled corpus was compared with the 150,595-word tokens in the larger children’s book corpus. The cross-corpora analysis focused on lexical diversity (type-token ratio, occurrence of unique words), word lengths (both phonological and orthographic lengths), and orthographic diversity (across the multiple orthographic units characteristic of the Kannada alphasyllabary).

### Lexical diversity

Type-token ratio (TTR) is an estimate of the ratio between the number of unique words present in a text (type) and the size of that text corpus (token). Since a TTR value changes with the size of the text corpus, the preferred approach is to plot TTR values as a function of token size and obtain a cumulative TTR. Here, we compared the rate of change in TTR values across multiple token sizes sampled from each text corpus. We used token sizes (number of words for an iteration of analysis) that increased linearly by a step size of 200 words, i.e., from 200 to 400 to 600, and further. To avoid a sampling bias within consecutive sample sets, the order of words from all the sentences in the corpus was shuffled each time, i.e., words for a given sample size were randomly selected. This step was repeated 20 times to obtain multiple estimates of both the TTR values and number of unique words. The identical procedure was applied to both the larger and the non-sequential small corpus.

Two further robustness checks were conducted. First, a comparison between the method of token selection for generating estimates (that is, random vs sequential sampling of token sets). The random sampling method is preferred for cumulative TTR estimations of lexical diversity over the use of consecutive words because of the expected repetition of the same content words within a single story. We examined the short corpus constructed using a non-sequential principle with a matched size of continuous text in the larger corpus that is then shuffled. Second, a comparison was made between two methods of constructing short corpora: a selective non-sequential vs. taking all sentences. Here the selective corpus of non-sequential texts was compared with a corpus matched for word token size but constructed with all sentences in a book. While the selective approach drew sentences from 402 books, the all-sentences approach required only seven books.

### Word length

Akshara and phoneme length per word were computed using the Automatized Linguistic Frequency Analyser (ALFA) developed by Agrawal and Nag (2021). ALFA is an open-source software designed to estimate the lingual properties of akshara languages. This software converts a text corpus into an array of words and then counts the number of akshara within each word to estimate akshara length. Kannada has a transparent orthography allowing for phonological lengths of words to be computed based on orthographic representation. Orthographic units for consonants without inherent vowel or a vowel diacritic (C) were counted as one phoneme, and the consonant with inherent vowel (Ca) or vowel diacritics (CV) were counted as two phonemes. Consonant clusters such as -kk*-* are counted as two phonemes and -tsy- as three. Thus, the word <kaage> ‘crow’ has four phonemes (k + aa + g + e) and <matsya> ‘fish’ has six (m+a+t+s+y+a). The performance of ALFA was validated against manual counting of akshara and phoneme lengths for 800 words. Concurrence in estimates was 100%.

### Orthographic diversity

In extensive writing systems such as the alphasyllabary of Kannada, it is expected that the range of akshara types will increase in older books (Nag, 2017). Eight akshara types (C, Ca, CV, CV with the nasal anuswara, CC, CCa, CCV and CCCV) were examined. Since the total number of words varied by book level, a direct comparison of the raw count is not appropriate. Instead, normalized frequency count (i.e., area under the histogram curve = 1) of each akshara type with the total number of akshara tokens within each book level was computed. To account for the variability in estimating frequency counts, 100 iterations were made with each akshara token set achieved with randomly selected words within each book level, and repeated replacement before the next random selection. The same method was used for both the larger and the non-sequential small corpus.

## Results

### Lexical diversity

**Fig. 1:**
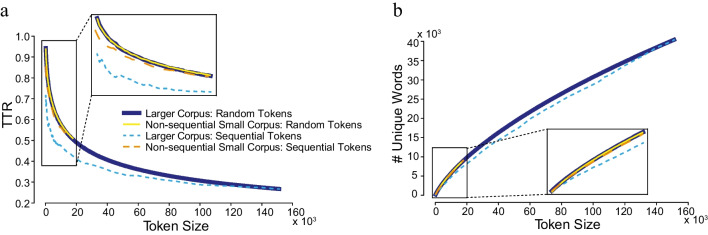
Cumulative curves for type-token (TTR) ratios (**a**) and unique words (**b**) per token size using random and sequential sampling approaches

A comparison of the larger and non-sequential small corpora on the measures of cumulative TTR and unique words using the random and sequential sampling approaches is shown in Fig. 1a, b. As expected, with an increase in token size, in both corpora, there was a gradual decrease in cumulative TTR scores but increase in the number of unique words. In terms of unique words, for the larger corpus, at a token size of 50,000 words, there were 19,052 unique words and at a token size of 150,000 words, unique word encounters increased to 40,191 words. A smaller corpus clearly cannot approximate these numbers. However, for comparative token sizes of 7500 and 15,000 words, the number of unique words found in the larger corpus was 4576 and 7864, respectively, while in the non-sequential small corpus it was 4620 and 7938, respectively. Although the cumulative TTR scores for these and other token sizes differed only marginally (see top panel, Table 1), the difference was statistically significant; *p* < 0.0005 using the *t* test.

**Table 1:** Lexical diversity, word length, and orthographic diversity comparison between the non-sequential small corpus and the larger corpus

	Non-sequential small corpus	Larger corpus
Lexical diversity (mean TTR (SD))		
7500-word token size ^1a^	0.616 (0.003)	0.610 (0.005)
15,000-word token size ^1a^	0.524 (0.002)	0.529 (0.004)
Word length (mean normalized frequency count (SD))		
Phoneme count ^1b^	7.00 (3.2)	6.96 (3.2)
Akshara count ^2^	3.32 (1.4)	3.3 (1.4)
Orthographic diversity (proportion of each akshara type in percentages)		
CV	45.60	45.80
Ca	33.50	33.20
CCV	9.10	9.10
CCa	6.00	6.10
CV+ ^3^	2.80	2.80
C	0.55	0.64
CC	0.05	0.05
CCCV	0.01	0.02

^1a, 1b^ The by-corpora difference is statistically significant; *p* < 0.0005 using *t* test^1a^; *p* = 0.045, unpaired *t* test ^1b^. ^2^ No significant difference; *p* = 0.07, unpaired *t* test. ^3^ CV + marker for the nasal anuswara or the aaha marker

For the first robustness test of the non-sequential small corpus, the method of selection of a token size for generating estimates had a bigger impact on the larger corpus compared to the small corpus. The non-sequentially chosen small corpus had more unique words and higher TTR scores than a comparable section of the larger corpus. The deviation in cumulative TTR values between the two corpora was significantly lower with random sampling compared to the sequential sampling approach (at the token size of 10,000 words, TTR_larger corpus_ – TTR_small corpus_ = 0.004 for randomly sampled token sets, and 0.09 for sequential sampled, both *p* < 0.00005 unpaired t-test).

Next, we questioned whether the protocol of sampling every tenth sentence from a larger number of books is more robust than taking fewer books but all sentences. For this, we compared the difference in cumulative TTR scores estimated with both a randomly and a sequentially selected token set. As expected, the non-sequential small corpus was less impacted by the method of token generation for estimating TTR scores: for example, at the token size of 10,000 words, the TTR_random_ – TTR_sequential_ difference for the non-sequential small corpus developed by extracting every tenth sentences was 0.017, and 0.037 for the same-sized corpus constructed by taking all sentences from a few books, *p* < 0.00005 (*t* test; see Supplementary Materials, Figure SM1).

### Word length

**Fig. 2:**
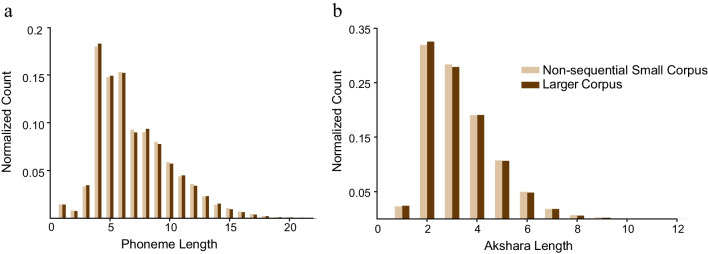
Normalized frequency counts of words by phoneme length (**a**) and akshara length (**b**)

For each word token in the larger and non-sequential small corpus, the phoneme and akshara lengths of words were computed (see middle panel, Table 1 for average values). The normalized frequency counts showed longer multi-phonemic and multi-akshara words in both corpora with a predominance of words of 4–7 phoneme lengths (Fig. 2a). Akshara lengths ranged from 2 to 10 units with words of 2 and 3 akshara being the most common in both corpora (Fig. 2b). On visual inspection, the normalized count distribution overlapped for phoneme frequency for the two corpora and yet their difference was statistically significant (*p* = 0.045, unpaired *t* test), indicating that the non-sequential small corpus was marginally different from the large corpus. However, there was no significant difference in akshara lengths in the two corpora (*p* = 0.07, unpaired *t* test) indicating that words in the two corpora were equivalent in orthographic word length.

### Orthographic diversity

**Fig. 3:**
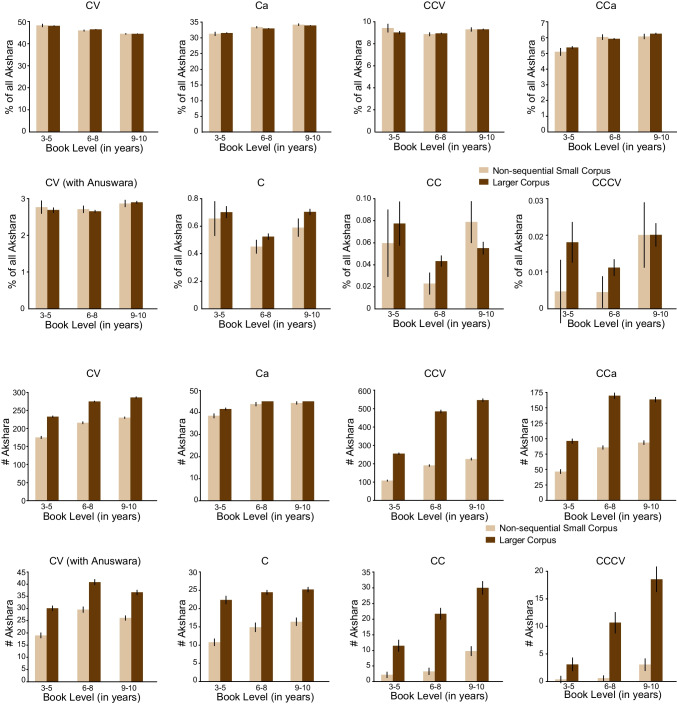
Proportion of orthographic units across book levels by akshara type (*top panel*) and number of unique akshara (*bottom panel*)

Examining orthographic representation in both corpora further, the average count of different akshara types was computed. The proportion of each akshara type within each corpus was similar across the two corpora (see bottom panel, Table 1). Of particular interest is rarely occurring orthographic units and whether the non-sequentially sampled small corpus includes these. The CV with the aha marker is a rarely occurring unit and this was found in the non-sequential small corpus. Further, the proportion of CV akshara decreased, and the Ca and CCa akshara increased with increase in book level (Fig. 3, top panel). Akshara diversity estimated as the number of unique akshara in each book level also showed the expected trend: akshara diversity increased with book level and was higher for the larger corpus compared to the smaller corpus (Fig. 3, bottom panel).

In summary, Study 1 shows no significant differences between the non-sequential small corpus and the larger corpus for orthographic length of words and the occurrence statistics for nine types of orthographic units with similar rate of occurrence including, notably, the rare types. Longer corpora will by definition have more instances of unique words and, in an extensive writing system such as the Kannada alphasyllabary, unique orthographic units. Significant differences were indeed found across multiple comparable token sizes. A strong claim of no statistically significant difference between the larger corpus and the shorter non-sequentially sampled corpus is therefore rejected. Despite this, the approximation of the small corpus to the large corpus for lexical and orthographic diversity and phonological length is noticeable and, for a matched corpus size, the cumulative TTR curves and proportion of unique lexical and orthographic occurrences are close. There is also a close association in the count of words of different phonological lengths. In addition, a step-wise change is expected in orthographic demand with increasing book level. This phenomenon is confirmed across multiple types of orthographic units and the developmental trend mirrors what is seen for each type in the larger corpus. In so much as these parameters are important metrics of language in child-directed print corpora, the findings demonstrate a robustness to the smaller selectively constructed corpus. In particular, the robustness is attributed to the corpora construction approach of using non-sequential text sourced from books stratified by book level. Finally, in a robustness check of lexical diversity in a corpus that is as small in size as the non-sequentially sampled small corpus but constructed using sequential sentences from a few books, the material is less diverse in the later, and more susceptible to the approach to token selection applied to estimate TTR scores. If a small corpus must be used for research and for applied purposes, then the use of a non-sequential sampling protocol for corpus construction makes the corpus statistics better.

## Study 2: Within-corpus analysis

With Study 1 indicating that the lexical, phonological, and orthographic statistics of the NSP-SCD approach to a child-directed print corpus are adequately equivalent to a larger corpus, we next examined the small corpus by parts of speech (PoS) – nouns, verbs, adjectives, adverbs, and pronouns – to find what changes and what stays the same across book levels. Proper names were tagged as such. Post-positions, conjunctions, quantifiers, demonstratives, particles, and residuals were tagged as ‘other’. For example, in the sentence, *I washed the red cup,* the word token *[I]* was tagged as a pronoun, *[washed]* as a verb, *[the]* as a determiner and hence as 'other', *[red]* as an adjective and *[cup]* as a noun.

The PoS tagging for each word token was done manually using the sentential context in which the word appeared. Tagging accuracy was verified by two raters who were proficient in the Kannada language and postgraduates in Language Sciences. Discrepancies in tagging were resolved following a two-step process: first, with two arbitrators who were experts in Language Sciences and by using Sridhar (1990) as the standard grammar, and second, with an expert in Kannada linguistics (Amritavalli, 2019; Jayaseelan & Amritavalli, 2017). The interrater agreement estimate before arbitration was at 88% for 20% of the corpus data (507 sentences comprising 1088 nouns, 930 verbs, 139 adjectives, 350 pronouns, and 170 adverbs).

### Measures

#### Lexical diversity, word length

Study 2 used the same procedures as described in Study 1. For lexical diversity, the cumulative TTR curve were plotted for each PoS category and the number of unique words were computed by PoS category for all books, and separately by book levels. For word length, the akshara and phoneme lengths were computed for words in each of the three book levels.

## Results

The developmental corpus statistics for sentences, words, and by PoS categories for the three book levels are given in Table 2.

**Table 2:** Number of sentences and words and percentage of words by parts of speech and book level

	Book level (Number of books)		
	3 to 5 years (64 books)	6 to 8 years (80 books)	9 to 10 years (36 books)
Sampled text			
Number of sentences	409	1260	1001
Range	1–63	1–125	2–125
Median (IQR)	3 (2)	6 (9.25)	15 (21.25)
Number of words	2047	7628	7712
Range	2–338	5–775	10–1015
Median (IQR)	18 (16.75)	41(71)	118.5 (183)
Parts of speech			
Noun (5770)	34.2%	34.18%	31.94%
Verb (4714)	27.5%	27.29%	26.82%
Pronoun (1794)	10.21%	10.34%	10.32%
Adverb (1020)	5.37%	5.5%	6.3%
Adjective (766)	4.74%	4.5%	4.2%
Other* (2458)	13. 92%	13.4%	14.91%
Proper noun (865)	4.05%	4.72%	5.47%

*Includes post-positions, conjunctions, quantifiers, demonstratives, particles, and residuals

### Lexical diversity by parts of speech

**Fig. 4:**
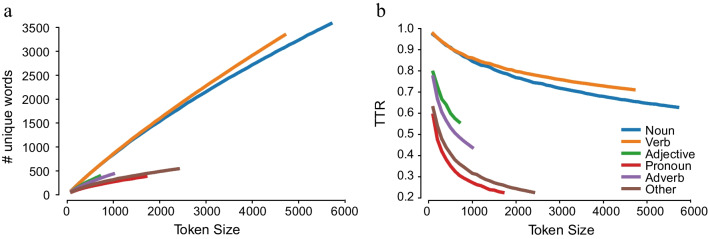
Cumulative curves for number of unique words (**a**) and type-token ratios (TTR) (**b**) per token size by parts of speech

The length of the curves for the occurrence of unique words (Fig. 4a) and the rate of decrease of cumulative TTR (Fig. 4b) for each PoS indicates that different parts of speech have different patterns of occurrence in the corpus. The highest number of unique words was with nouns followed by verbs, pronouns, adverbs, and adjectives, respectively. Also, the rate of increase of unique words differed by PoS category for a given token size. For instance, for a randomly picked set of 1000 pronouns there can be only about 250 unique pronouns, whereas a similarly randomly picked 1000 nouns had at least 700 unique nouns. Not surprisingly then, the TTR values were higher for the open-class category of nouns as compared to the closed-class category of pronouns.

**Fig. 5:**
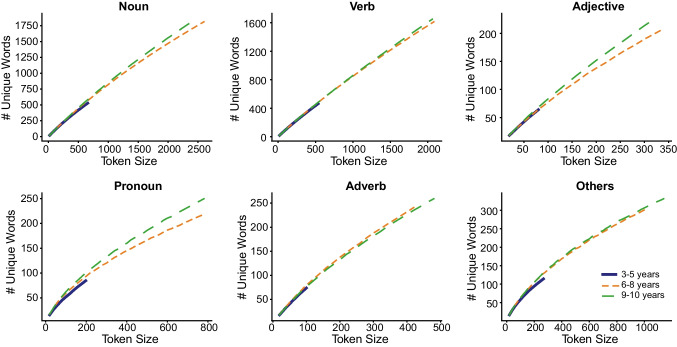
Cumulative curves for number of unique words per token size by parts of speech and book level

**Fig. 6:**
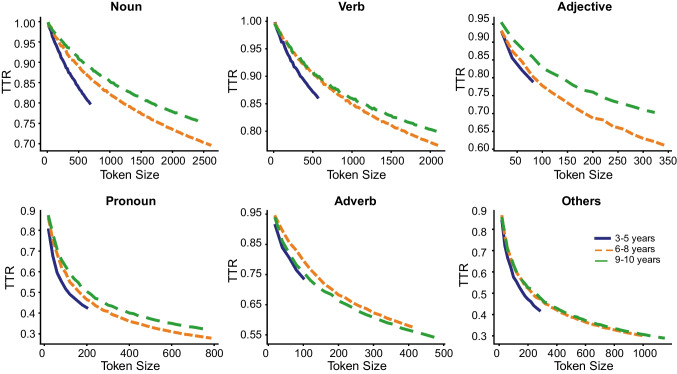
Cumulative curves for type-token ratios per token size by parts of speech and book level

In the next developmental enquiry of the corpus data, we examined if books designed for older children were lexically more diverse by plotting for each PoS category, and by book level, the number of unique words (Fig. 5) and the curves of the cumulative TTR scores (Fig. 6). The different lengths of curve indicate differences in the corpus size for each book level. The number of unique words increased by book level with the lowest number of unique words (at a given token size) for the 3-to-5-years book level, compared to the higher book levels. The rate of change of the TTR values indicates that the TTR values decreased more rapidly in the 3-to-5-years book level, and more for nouns and verbs. The decrease in TTR value is indicative of more repetition of words used in books for the early years, and this is especially with nouns.

### Word lengths by book levels

The mean phoneme lengths were close across book levels yet significantly larger in the higher book levels; (Mean_phoneme length_ = 6.7 (3), 7 (3.1), and 7.1 (3.4) for the 3-to-5-, 6-to-8- and 9-to-10-years book level with all pair-wise differences statistically significant (*p* < 0.05, unpaired *t* test). Similarly, words in higher book levels contained more akshara; (Mean_akshara length_ = 3.2 (1.3), 3.3 (1.4) and 3.4 (1.5) for 3–5, 6–8- and 9–10-years book levels, respectively. All pair-wise differences between book levels were statistically significant (*p* < 0.05, unpaired *t* test).

In summary, the number of words and sentences increased in books for older age bands. Alongside this, while unique word encounters expectedly increased with an increase in token size, the language-specific trends help characterize one psycholinguistic characteristic of Kannada: nouns and verbs were more prominent across all book levels compared to pronouns, adverbs, and adjectives. The mean word length, both phonological and orthographic lengths, increased with an increase in book level replicating the finding from Study 1 that older children reading older books encounter longer phoneme and akshara strings compared to younger children who are primarily exposed to younger books.

## Discussion

The present study reports a systematic enquiry of a non-sequential sampling protocol for small corpora development, the NSP-SCD. A cross-corpora analysis examines the psycholinguistic parameters on which such a selectively constructed corpus is either equivalent to or approximates a larger corpus. First, we found the proposed protocol provided a corpus that was more robust than a similar-sized small corpus using continuous texts where all sentences from a small number of books were taken; an approach that may be intuitively adopted in the absence of corpus data. Here, the hypothesis was that the NSP-SCD having controlled for the tendency of sequential text to provide more word repetitions due to shared topic coverage, would be immune to method of selection of token sizes to estimate type-token ratios while the all-sentences approach would not. This hypothesis is accepted or, more accurately, failed to be rejected. Second, we found equivalence in word-level characteristics between the non-sequential small corpus and the larger corpus. There were no significant differences on word lengths counted as number of orthographic units and the nature of orthographic representation measured through nine types of orthographic units. For word length when counted as number of phonemes per word, the corpora though close were still significantly different. Similarly, for metrics that are sensitive to token size – number of unique instances, type-token ratios – the selective corpus was not equivalent. This was expected since larger corpora by nature will have more unique words. However, even while the statistically significant difference was expected, it is important to note that the non-sequentially sampled small corpus still produced corpus characteristics that approximated the larger corpus well. Finally, cross-corpora equivalence was assessed through a developmental analysis comparing orthographic diversity across book levels. Equivalence was found across the nine types of orthographic units with the non-sequential small corpus retaining equivalence even on the less frequently occurring units. The sensitivity of the NSP-SCD to pick up even rarely occurring orthographic features is taken as a further indication of its usefulness as a research tool.

The second research question was developmental in nature with the hypothesis that there will be an increase in text demand in older books. This was confirmed in a within-corpus analysis. The median number of words and sentences increased by book level with a bigger jump between books for 3-to-5-year-olds and 6-to-10-year-olds. At the lexical level, there was an increase in the number of unique words and these were predominantly in the noun and verb categories. At the phonological level, word lengths increased marginally although even books for the youngest 3-to-5-level had words with a phoneme count of up to 20. Finally, orthographic diversity increased in older books in the form of more rare and complex akshara types appearing alongside the common and early appearing simple akshara (the CCa, CCV, CCCV akshara and the Ca, CV akshara, respectively). While the proportion of Ca and CCa akshara increased with an increase in book level, the occurrence of CV akhara units decreased with an increase in book level. This later finding is unexpected. More analysis is needed to examine different CV representations and how they each map to Kannada phonology particularly when words have more consonant clusters.

Together, the finding of equivalence and promising approximations have implications for the construction of small corpora. It is expected that smaller corpus sizes will not provide a clear and actual representation of a language’s psycholinguistic properties, particularly when the corpus is below a certain threshold. The reason for this is understood in the earlier mentioned Herdan-Heaps law about the relation between tokens and types, with the relation changing non-linearly with sample sizes. The number of unique words increases as the number of word tokens increase but at a slower rate as more words are added to the sample. We show that a non-sequential sampling protocol can to a certain extent manage the vulnerabilities of small-sized corpora to produce useful and effective estimates on psycholinguistics properties of interest to child language research.

A further point is in relation to diversity estimates (TTR estimations). In line with a trend in the literature, we computed the lexical diversity and orthographic diversity metrics using a multiple random sampling approach and linearly increasing token sizes by a step-size of 200 words (Dawson et al., 2021; Montag et al., 2015, 2018). However, a comparison of 20 estimates with hundred estimates showed little change in cumulative TTR estimates. It is likely that the non-sequential small corpus is less vulnerable to various possible approaches to token selection to compute TTR as the corpus is already constructed with non-sequential texts. An unexpected positive from this finding is that we could apply a cost-benefit analysis on the computation time involved and limit our procedures to twenty estimates. Such cost–benefit analyses are especially relevant for boosting research in understudied languages.

What did we learn about Kannada, the language of this study? Kannada uses a variety of the Indic writing system. Kannada, like the other akshara orthographies of south and southeast Asia, has an inventory size of more than 600 symbols that shapes orthography learning in ways quite different from the learning of the more extensive Chinese systems and the substantially contained alphabetic symbol sets (Nag, 2017). Given the sheer variety of orthographic units the writing system uses, the fidelity of orthographic representation in the non-sequentially sampled small corpus is therefore especially of interest. The cross-corpora analysis confirms that orthographic representation in the non-sequential small corpus closely mirrors the larger corpus and that this is extended to the easy to miss, less frequently occurring types. At the word level, the dominant word lengths in Kannada are between 4 and 6 phonemes and 2 and 4 akshara, and there are more nouns, verbs, and pronouns in book language compared to adverbs and adjectives. Unlike English but like Turkish, the occurrence of adverbs is more in Kannada than adjectives while there are more conjunctions in English and Turkish compared to Kannada (Aydin, 2019; Dawson et al., 2021). The corpus data extends insights from behavioral data about akshara learning (Nag, 2007; Padakannaya et al., 2015; Tiwari et al., 2021) – some akshara types are rare and later appearing and the trends across book levels map well with the converging finding that learning of the akshara system continues well past middle school (the age band of the older books in the corpora we studied).

Finally, what constraints might the non-sequential sampling protocol place on corpus development? First, non-sequential sentences could result in an over-representation of certain word categories such as pronouns. One possibility for future research would be to compare the characteristics of small corpora constructed through a pseudo-random selection of sentences as against the non-sequential protocol of every tenth sentence. In the pseudo-random approach, sentences would be added iteratively to the selective corpus such that every new sentence added contains at least one new word not appearing elsewhere in the corpus. Second, a limitation of the study is that the parts of speech analysis was done with only a section of the non-sequential small corpus due to the sheer time and resource needed for manual tagging. It is therefore difficult to infer to what extent corpus statistics for different parts of speech are an actual representation of the written language complexity as captured in the larger corpus. Finally, an important area of investigation in child language and learning in understudied languages is the role played by contextual diversity. The study of contextual details of words and orthographic units, their transitional frequencies and conditional co-occurrences are important next steps to examine because the variety that stems from exposure in diverse settings can support learning, most notably through stronger generalizations (Raviv et al., 2022), and this principle of learning is also true for language learning (Hiebert, 2005; Hsiao & Nation, 2018). Specific to this paper, it is important to understand how a non-sequential sampling protocol impacts the variability in contexts where words and symbols appear. Also unclear is the extent to which the rich semantic networks and patterns of occurrence and transitional probabilities of words seen in large corpora is protected with non-sequential sampling. The data from our analyses (Study 1) for orthographic diversity suggests that at least for units of a writing system, the non-sequential sampling protocol is promising for representation of a wide range of orthographic units, what remains to be seen is whether diversity statistics remain similar to a large corpus.

In conclusion, our study demonstrates the characteristics and levels of robustness of a small sized child-directed corpus and proposes that the non-sequential sampling protocol may be used as a tool to systematically support child language research in understudied languages. The growing availability of children’s books in understudied languages internationally offers the opportunity. For cognitive science, the resource of a robust small corpus promises to support the development of better experiments and psychometrically sound measures. For education, the resources offer a developmental catalogue of rich language that could inform intervention planning that may support oral language and literacy development.

### Supplementary information

ESM 1 (DOCX 321 kb)
