# Supplementary material for: NSP-SCD: A corpus construction protocol for child-directed print in understudied languages
Source: Behav Res Methods. 2024 Feb 15;56(4):2751–64. doi: 10.3758/s13428-024-02339-x (PMC11133114; doi:10.3758/s13428-024-02339-x)

**Figure SM1**

*Comparing Corpus Sampling Strategies at Equal Lengths from the Larger Corpus and the Non-sequential Small Corpus*


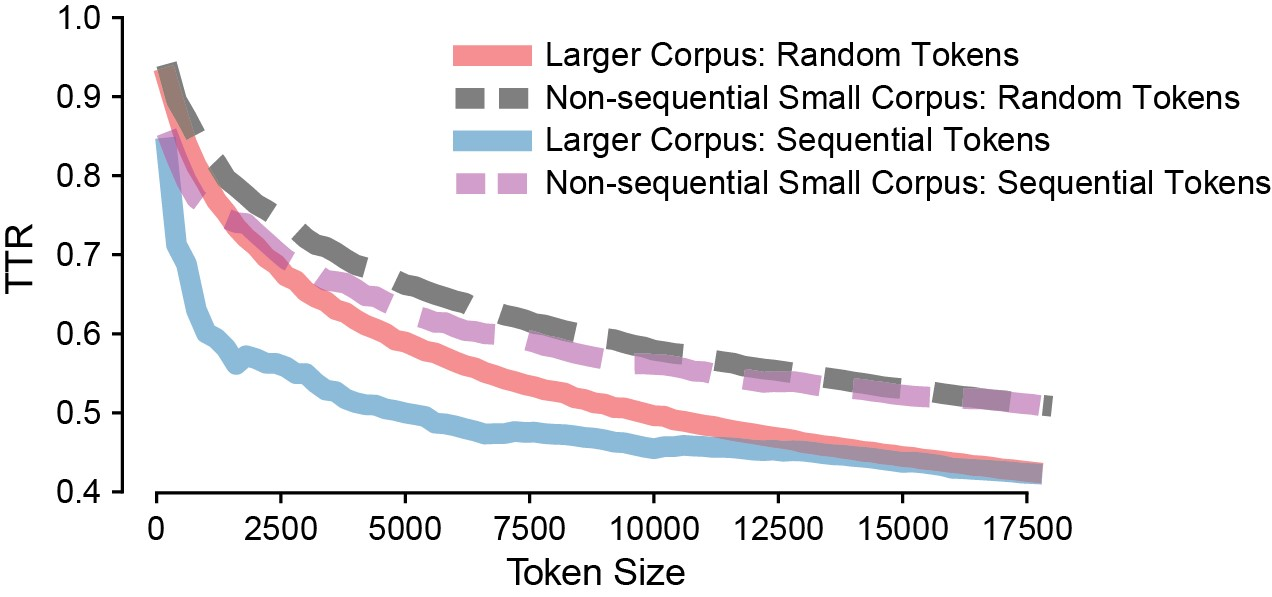

Supplement: Supplementary file 1 — (DOCX 321 kb) [file 13428_2024_2339_MOESM1_ESM.docx]
